# The Use of 3D Technology in the Management of Residual Asymmetry following Orthognathic Surgery: A Case Report

**DOI:** 10.3390/healthcare11152172

**Published:** 2023-07-31

**Authors:** Neculai Onică, Cezara Andreea Onică, Dana Gabriela Budală, Gabriela Luminița Gelețu, Mihail Balan, Elena-Raluca Baciu, Alice Murariu, Mihaela Pertea

**Affiliations:** 1 Independent Researcher, 700612 Iasi, Romania; nicuonica@yahoo.com (N.O.); dr.cezaraonica@gmail.com (C.A.O.); 2 Department of Implantology, Removable Dentures, Dental Technology, Faculty of Dental Medicine, University of Medicine and Pharmacy “Grigore T. Popa”, 700115 Iasi, Romania; elena.baciu@umfiasi.ro; 3 Department of Surgery, Faculty of Dental Medicine, University of Medicine and Pharmacy “Grigore T. Popa”, 700115 Iasi, Romania; mihail.balan@umfiasi.ro (M.B.); alice.murariu@umfiasi.ro (A.M.); 4 Department of Plastic Surgery, Faculty of Medicine, University of Medicine and Pharmacy “Grigore T. Popa”, 700115 Iasi, Romania; mihaela.pertea@umfiasi.ro

**Keywords:** orthognathic surgery, computer-assisted design, computer-aided manufacturing, polymethyl methacrylate, facial asymmetry

## Abstract

The purpose of this case report was to present the aesthetic result of the reconstruction of facial residual asymmetry after orthognathic surgery using a patient-specific three-dimensional (3D) mold and a custom-made polymethyl methacrylate implant. Through computer-aided design (CAD), the healthy contralateral side of the mandible was superimposed onto the side with the defect. Exocad Gallway (exocad GmbH, Darmstadt, Germany) was used to design the patient-specific implants (PSIs) of the right mandibular angle. Next, the implant mold was created using the Meshmixer software (Version 3.5, Autodesk Inc., San Rafael, CA, USA) and fabricated using additive manufacturing. During the surgical procedure, the patient-specific implant (PSI) was cast inside the resin mold using Simplex P bone cement (Stryker, Mahwah, NJ, USA). The implant was fixed using three screws. Combining both indirect (involving the dental laboratory) and direct (with surgical intervention) approaches, this innovative hybrid method, which incorporates both computer-aided design and additive manufacturing (AM), not only enhanced facial aesthetics, functional rehabilitation, and patient quality of life but also mitigated the potential risks linked to conventional grafting methods.

## 1. Introduction

The aim of orthognathic surgery (OGS) is to provide bone and soft tissues with a symmetrical and functional appearance. Kronmiller et al. [1] defined symmetry as the exact sameness of the right and left sides of the face. Numerous studies have shown that hard and soft tissues alter in the wake of orthognathic surgery [2,3,4]. However, investigators’ accounts of the restoration of bone symmetry frequently highlight a lack of sufficient soft tissue symmetry. The mandatory surgical separation of the facial muscles as a result of severe bone deformities influences this perception. Thus, there are substantial soft tissue volume deficits on both the right and left sides of the face [5]. In these instances, additional surgical procedures to change the amount of the skeletal and soft tissue components on both sides are required to achieve better symmetry [6].

The benefits of orthognathic surgery based on patient-specific implants (PSIs) are well explained, and this technique has been successfully used for the last few years [7,8]. Facial implants can also be employed in augmentative surgeries to appropriately restore bony abnormalities or malpositions [9,10].

There are two main categories of grafting methods used to address mandibular defects: autogenous and alloplastic. Due to postoperative infection, donor site morbidity, and unpredictable bone resorption results, autogenous bone grafts are discouraged.

Initially, commonly used preformed stock alloplastic materials like porous polyethylene, polytetrafluoroethylene, and silicone could be adjusted to match a defect during surgery, but their disadvantages included empty spaces, migration risks, and infection due to shape mismatch with the underlying bone [11,12].

Polymer powder and a liquid monomer are combined to produce polymethyl methacrylate [13]. Polymer powder is composed of re-polymerized polymethyl methacrylate beads, a radiopaque component (such as BaSO_4_ or ZrO_2_ particles), and an initiator (benzoyl peroxide) that starts the polymerization reaction. Along with an antibiotic, the powder also includes barium sulfate (10–15 weight percentage) as a radio-pacifying agent for improved vision during clinical usage [14]. A minor quantity of hydroquinone, an activator (N, dimethyl-para-toluidine) that encourages radical formation, and methyl methacrylate monomer make up the liquid component. To avoid early polymerization during storage, the liquid component also contains a reaction inhibitor or stabilizer [13].

The advantages of PMMA include stability, high biocompatibility, low radiopacity, strong resistance to functional stress, easy handling, and low costs [7,15,16]. Lye et al. [17] define PMMA as the most suitable option for potential use in a mandibular endoprosthetic system. Regardless of its benefits, however, it presents several challenges in terms of exothermic reactions, toxic gases created during the mixing process, and rare allergic reactions that must be addressed [18,19].

Despite the limited number of clinical reports on the application of PSIs for mandibular contour restorations [2,5,7,9,20], this paper aimed to present a fully digitized workflow to create template-molded PSIs using PMMA bone cement during surgery.

## 2. Case Report

**Figure 1:**
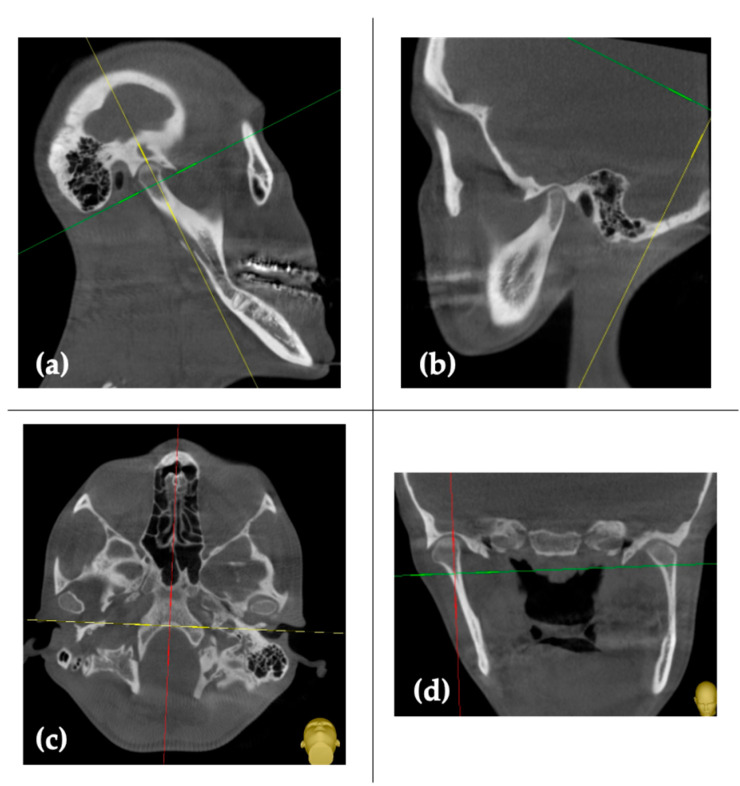
Pre-surgical CT scans: (**a**,**b**) sagittal sections; (**c**) axial section; and (**d**) coronal section.

A 23-year-old woman presented with skeletal class III, associated with a condylar volume asymmetry between the left and right sides (Figure 1a–d).

**Figure 2:**
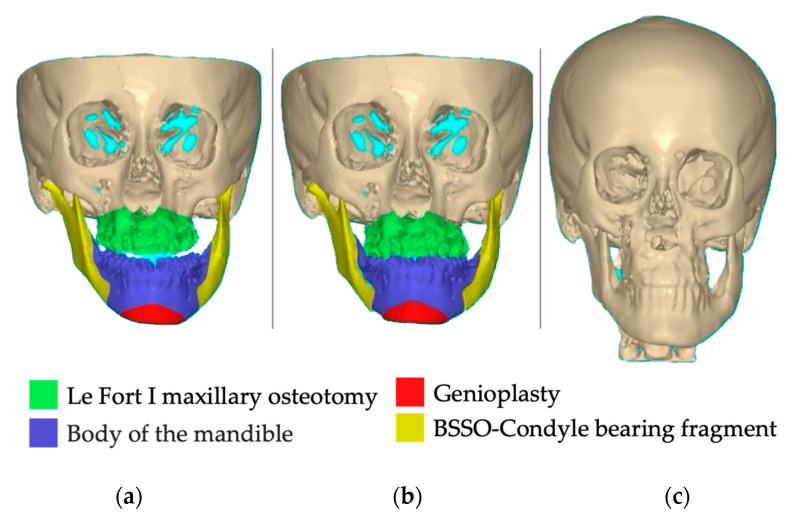
Pre-surgical computer-assisted virtual simulation created using NemoFab (Nemotec, Madrid, Spain): (**a**) frontal view of patient skull; (**b**) frontal view of planned final reposition; and (**c**) 1-year follow-up CBCT control.

She had undergone orthognathic surgery twelve months previously, which involved bilateral sagittal split osteotomy, genioplasty, and Le Fort I osteotomy. The surgery resulted in a significant improvement in the severe asymmetry in the lower third of her face. Despite the positive outcome achieved by our team, the patient returned to our dental office for further treatment (Figure 2a,b), as additional refinement was still required to address the remaining asymmetry. She had no major family history of illnesses or systemic conditions.

One year after OGS, three-dimensional face images were acquired using a computed tomography (CT) scanner (SOMATOM sensation 10, Siemens, Munich, Germany) with the following settings: 120 kVp tube voltage; 80 mAs tube current; and 0.6 mm slice thickness (Figure 2c).

Upon receiving a comprehensive explanation encompassing the diagnosis, prognosis with and without treatment, specific therapeutic measures, and the advantages, procedure-specific risks, and potential side effects associated with the treatment, the patient granted her informed consent.

### 2.1. PSI’s Mold Design and Fabrication

**Figure 3:**
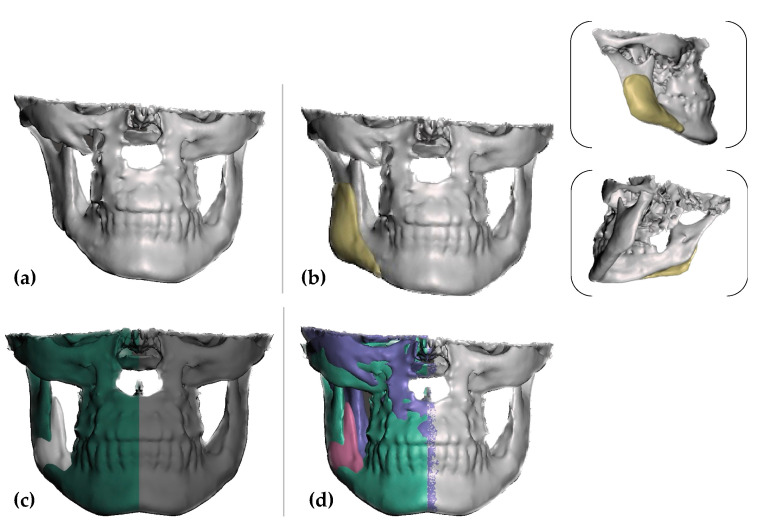
Virtual images of the designed patient-specific implant using Exoplan 3.0 Dicom Viewer (exocad GmbH, Darmstadt, Germany): (**a**) initial situation, (**b**) PSI frontal view; and (**c**,**d**) mirroring the left side (healthy contralateral).

The data were exported in a Digital Imaging and Communications in Medicine format (DICOM) and then converted to standard tessellation files (STL) using the Exoplan 3.0 Dicom Viewer (exocad GmbH, Darmstadt, Germany). The PSI’s design was created using Exocad Gallway (exocad GmbH, Darmstadt, Germany) on the right mandibular angle, using the normal, healthy contralateral as the model (Figure 3a–c).

**Figure 4:**
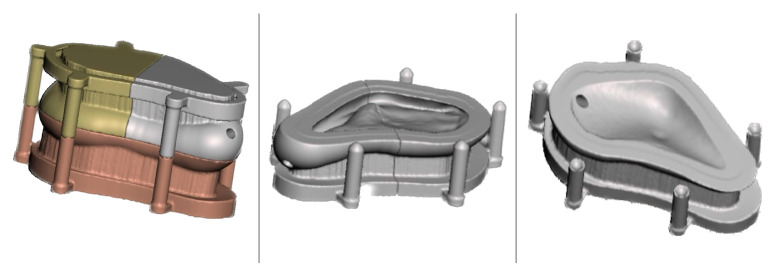
Virtual images of the designed mold using Meshmixer software (Version 3.5, Autodesk Inc., San Rafael, CA, USA).

The Meshmixer software (Version 3.5, Autodesk Inc., San Rafael, CA, USA) was used to smooth the surface and create the implant mold (Figure 4).

The STL file of the 3D-designed mold was sent to an ASIGA 3D MAX UV printer (ASIGA, Alexandria, NSW, Australia) to be fabricated using Phrozen Water-Washable Dental Model 3D Printer Resin (Phrozen Technology, Hsin-chu, Taiwan). Prior to surgery, a 25 min immersion in Gigasept PAA (Schülke & Mayr GmbH, Norderstedt, Germany) sterilization was carried out.

**Figure 5:**
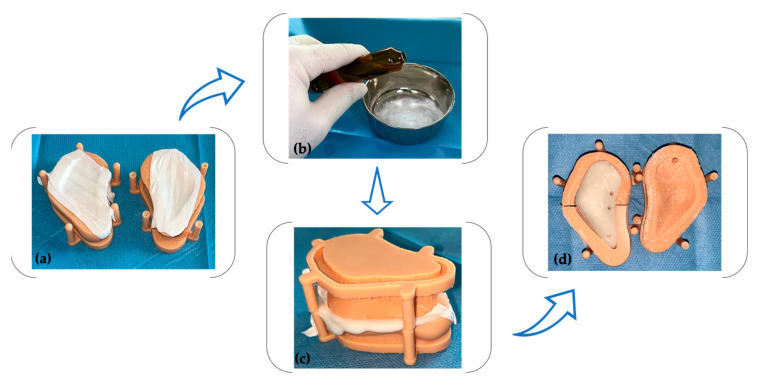
The PSI fabrication process involved the following steps: (**a**) isolation of the mold using a sterile Teflon band; (**b**) mixing the monomer and polymer components; (**c**) allowing for a setting period; and (**d**) trimming and drilling the implant.

During the surgical procedure, the bone cement (Surgical Simplex P, Stryker, Mahwah, NJ, USA) was unpacked and utilized. The monomer and polymer components were mixed in accordance with the manufacturer’s instructions (Figure 5b), and the mixture was subsequently injected into a previously isolated mold using a sterile Teflon band (Figure 5a). Following an 8 min polymerization period (Figure 5c), the cast was opened, and the PSI (patient-specific implant) was extracted. Minor smoothing and drilling were conducted (Figure 5d) using a tungsten carbide bur (H251EQ, Komet Dental, Lemgo, Germany) and a 2.2 mm drill (Medicon eG, Tuttlingen, Germany).

To ensure complete cooling of the implant (to counteract the exothermic polymerization reaction effect), it was immersed in Betadine (Egis Pharmaceutical PLC, Budapest, Hungary) for an additional 10 min. The PMMA implant used in this case had a minimum thickness of 1 mm in the upper and anterior poles and a maximum thickness of 7 mm in the posterior and lower pole.

### 2.2. Surgical Procedure

The blood test results of the individual were within the expected parameters. During the initial phase, the individual was administered a non-steroidal analgesic (Ketorol, Dr. Reddy’s Laboratories, București, Romania) through an intravenous route. Additionally, 2 g of amoxicillin was administered as a loading dose.

To ensure the proper sterilization of the surgical area, the patient performed a 1 min rinse using Curasept (Curaden AG, Kriens, Switzerland). Then, a sterile surgical drape was utilized to disinfect and cover the surgical site.

Inducing local anesthesia involved the utilization of 4% articaine with epinephrine 1:100,000 (Ubistein, 3M ESPE, St. Paul, MN, USA). To locate the issue, customary vestibular incision and subperiosteal dissection were utilized.

The PSI was firmly seated after sufficient inferior and posterior side dissection. The PSI was applied directly and fastened with three 2.0/9 mm mini-screws obtained from Medicon (Medicon eG, Tuttlingen, Germany). The first two screws were positioned in the retromolar area, approximately 1 cm apart, and inserted with a tilted trajectory in the buccal–lingual direction, with a distal orientation, effectively securing the implant in the oblique ridge region. The third screw was placed in the lower border, specifically on the mesial aspect of the second molar, with as much of a horizontal orientation as possible.

During the postoperative period, the patient’s treatment plan included a prescription for amoxicillin and clavulanic acid (Augmentin, Glaxo Wellcome, Mayenne, France). The prescribed dosage was 2 g (1 g taken orally twice a day) to be taken for six days. Furthermore, the patient was advised not to brush the surgical site for a period of three weeks, to consume soft foods, and to maintain good oral hygiene practices.

**Figure 6:**
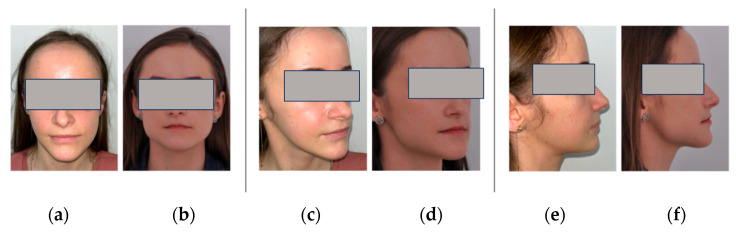
The extraoral images of patient frontal, semi-profile, and profile: (**a**,**c**,**e**) preoperative; (**b**,**d**,**f**) 1 month postoperative.

During the first month, the patient attended weekly examinations, followed by monthly follow-ups. No instances of exposure or infection were observed. Following the procedure, the patient did not experience any particular discomfort, such as pain or increased numbness in the jaw and lower lip, and she was satisfied with the significant improvement in facial appearance (Figure 6a–c).

## 3. Discussion

The link between condylar and craniofacial asymmetries is an intriguing subject in the field of dentistry and maxillofacial surgery. Recent studies have explored the relationships between various asymmetries and how they might be connected [21,22].

Modern CAD software facilitates the act of mirroring, enhancing symmetry, and enabling the precise alignment of implants with the contralateral side [18,19,23,24]. Nevertheless, it should be noted that this mirroring process has been associated with overcorrection. Despite this challenge, we considered it a reasonable compromise in our approach.

In recent years, PSI bone augmentations have emerged as a beneficial approach for treating facial asymmetries following orthognathic surgery [25,26]. Compared with patients with non-laterognathic dysgnathia and symmetrical craniofacial deformities, achieving symmetry in patients with laterognathic disorders, whether syndromic or not, is far more difficult [27].

The use of PSIs for maxillofacial reconstruction features predictable outcomes, eliminates the usual complications seen in non-custom-made implants, and boasts excellent patient satisfaction. Its main drawback is the high cost.

PSIs are a proven method of facial skeleton rebuilding, and their usage in orthognathic surgery is gradually gaining popularity. The repeatability, simplicity, and low complication rates of PSIs in nonsyndromic craniofacial patients have been acknowledged in a number of studies; however, data on orthognathic patients are rather restricted due to the recent use of PSIs. In our case report, we observed no complications related to custom-made PSIs.

It is important to note that while orthognathic surgery only requires an accuracy of 1.0 mm in linear distance deviation and a 2–4° angular deviation, occlusal fine tuning is achieved postoperatively through orthodontic treatment [28]. Occlusal rehabilitation necessitates a precision of 20–40 µm [29]. Since no viscerocranium is entirely symmetrical in nature [30], facial asymmetry of 1.0–2.0 mm is barely noticeable. When it comes to individuals with laterognathy, especially those with severe craniofacial abnormalities, the question arises of whether contemporary surgical planning and execution methods can accomplish such symmetry.

There is a wide range of materials available for bone regeneration and as substitutes for repairing bone defects, particularly in cranial bones. Commonly utilized materials for this purpose include metals like titanium, tantalum, and their alloys, as well as magnesium and its alloys. In addition to metals, ceramics, polymethyl methacrylate, poly-L-lactic acid (PLA), polylactide (PLLA), and polyetheretherketone (PEEK) are also frequently employed as bone substitutes.

Titanium (Ti), which may be created utilizing milling or three-dimensional (3D) printing technologies to generate a patient-specific implant (PSI), is one of the most biocompatible materials. Titanium implants have been utilized for internal maxillomandibular fixations and as dental implants for several decades since this biocompatible inert material has an inherent tendency toward osseointegration with the surrounding bone. Its proven biocompatibility makes it the material of choice for internal fixation and reconstruction in the craniofacial region. As a result, titanium 3D PSIs provide hope for the future of patients with this issue [31].

Another prominent alloplastic substance is PEEK, which has a number of appealing qualities such as strength, durability, resilience to environmental conditions, and a decreased infection rate [32]. PEEK bone augmentations have been found to be useful in the treatment of facial asymmetry in recent years. Because of this, additional improvements in facial symmetry following orthognathic surgery have been accomplished [25,33,34], including significant enhancements in soft-tissue symmetry [35]. PEEK augmentations have no morbidity at the donor site and are always an option [36], which sets them apart from autologous replacement procedures. The benefits of a PSI-based orthognathic operation with the insertion of PEEK bone augmentations are undeniable, but the technique is not without its drawbacks. The introduction of a foreign body can lead to infection [37]. Antibiotic shielding techniques, however, can mitigate this threat [38]. Larger augmentations increase the risk of complications such as infection and implant exposure or recurrence [39].

Polymethylmethacrylate (PMMA) cement reconstruction has demonstrable durability and a manageable learning curve when it comes to restoring the natural contour and shape of the skull [12,15]. According to Slimani et al. [16] this method is preferable since it is easy to implement, efficient, and flexible enough to accommodate defects of varying sizes. Initially tested on monkeys, the procedure was eventually used on humans in 1941 to correct cranial bone abnormalities [40]. Besides its use in neurosurgery, PMMA cement is extensively employed in dentistry, maxillofacial surgery, traumatology, and orthopedics. Due to its capacity to promote cell ingrowth, it can serve as a substitute for natural bone. The attachment of cells to this substance and their growth within its pores would result in good integration with biological tissues [40,41,42].

To ensure consistent graft robustness without thin sections that could lead to potential fracture sites, it is crucial to achieve the proper thickness of PMMA cement. This provides visually pleasing results and reduces the risk of complications such as wound dehiscence, infection, and the need for revision procedures resulting from tensionless wound closure. Previous studies have reported cytotoxic damage and thermal tissue damage caused by PMMA implants directly molded on the lesion due to the exothermic polymerization reaction during hardening [25]. However, the procedure described in this paper, utilizing indirect and remote molding, poses no harm to sensitive tissues.

To generate maximum surface friction and maintain long-term stability, precise positioning of the targeted PSI is of the highest significance [18]. Improper positioning of the PSI may damage the inferior alveolar nerve during screw fixation and can result in the implant’s edge being perceptible [43]. In cases where the buccal vestibule has significant fibrosis or scarring from prior surgery, a submandibular approach may be considered [18]. However, intraoral techniques are generally recommended to minimize the risk of extraoral scarring in aesthetic cases [23,44]. Based on our clinical experience, with a proper incision and sufficient reflection of the periosteum, the implant can be inserted with ease, avoiding unnecessary contact with the oral mucosa. By employing bimanual manipulation—intraorally with forceps and extra-orally with the fingers of the left hand behind the posterior border of the ascending ramus and lower border of the mandible—the implant is smoothly seated and effortlessly finds its proper position.

Mechanical fixation with screws is the most common method used to maintain PSI stability after surgery. The size of the implant might affect the total number of screws used. However, further clinical studies involving larger patient cohorts and finite element analysis are necessary to determine the optimal number of screws. In the case presented, the PSI was fixed using three screws.

Despite the relatively short follow-up period (6 months), it is important to note that the patient did not experience any complications associated with PSI reconstruction, and there was no evidence of infection. The wound healing was uneventful. Consistent with these results, earlier research has determined that infection rates during maxillofacial PSI repair are extremely low (between 7.7 and 14.3%) [6,10,24]. Based on Alasseri et al.’s [45] experience with non-custom-made implants, postoperative infections are typically seen within the first few weeks and rarely occur after one month.

One limitation of this technique lies in the requirement for expertise in utilizing the software that generates both the PSI and the PSI molded cast. Our case report confirmed the excellent viability and low cost of using a PSI molded cast during surgery. This approach has the potential to extend accessibility to a broader range of patients. Furthermore, a PSI acquired intraoperatively from a molded cast ensures an accurate fit and optimal functional and aesthetic outcome.

## 4. Conclusions

In conclusion, our case demonstrates that intraoperative-template-molded osteoplasty using PMMA is a safe, sustainable, efficient, and cost-effective technique for accurately recreating a patient’s natural bone thickness. By employing a single-stage repair method, we observed enhanced functional, aesthetic, and psychological results while also minimizing discomfort for a substantial number of patients. Our findings highlight the potential benefits and effectiveness of this approach in the specific context of our case.

## Data Availability

Not applicable.
